# No Correlation of hsa-miR-148a with Expression of *PXR* or *CYP3A4* in Human Livers from Chinese Han Population

**DOI:** 10.1371/journal.pone.0059141

**Published:** 2013-03-20

**Authors:** Zhiyun Wei, Mingjie Chen, Yiting Zhang, Xiaofei Wang, Songshan Jiang, Yang Wang, Xi Wu, Shengying Qin, Lin He, Lirong Zhang, Qinghe Xing

**Affiliations:** 1 Children’s Hospital and Institutes of Biomedical Sciences, Fudan University, Shanghai, People’s Republic of China; 2 Bio-X Institutes, Key Laboratory for the Genetics of Developmental and Neuropsychiatric Disorders (Ministry of Education), Shanghai Jiao Tong University, Shanghai, People’s Republic of China; 3 Department of Pharmacology, School of Medicine, Zhengzhou University, Zhengzhou, People’s Republic of China; 4 State Key Laboratory of Biocontrol and MOE Key Laboratory of Gene Engineering, School of Life Sciences, Sun Yat-Sen University, Guangzhou, People’s Republic of China; University of Birmingham, United Kingdom

## Abstract

There is a huge variability of hepatic CYP3A4 level in human populations, which was believed to contribute to different responses to drugs among individuals. Transcription of *CYP3A4* was regulated by transcription factors such as pregnane X receptor (PXR). MiRNA hsa-miR-148a was previously reported to influence *PXR* expression in HepG2 cells and in Japanese populations. In this study, we conducted a similar correlation study in Chinese Han population (N = 24). No significant correlation of hsa-miR-148a was found with *PXR* expression or *CYP3A4* expression. Our results suggest that hsa-miR-148a does not play a major role in the regulation of *PXR* or *CYP3A4* expression in human livers from Chinese Han population.

## Introduction

Cytochrome P450 3A4 (CYP3A4), the most abundant hepatic and intestinal member of cytochrome P450 superfamily [1], contributes to the metabolism of over half of the current prescription drugs [2], [3]. However, the CYP3A4 level has been reported to vary a lot (as great as 50-fold) in the general population, which cannot be explained by genetic polymorphisms alone [4]. Such huge inter-individual differences of CYP3A4 levels could explain the striking individual differences in drug responses, in terms of both therapeutic effects and adverse side effects [5]. Therefore, the regulation mechanism of *CYP3A4* expression became a research hotspot in pharmacokinetics [6], [7].

Previously, Takagi et al. reported that the protein level of pregnane X receptor (PXR), which is a major transcription factor of *CYP3A4* [6], [8], [9], correlated significantly with the CYP3A4 mRNA and protein levels in Japanese population [10]. After *in vitro* investigation with reporter system, they further found that the translational efficiency of PXR (PXR protein/PXR mRNA ratio) was inversely correlated with the expression level of hsa-miR-148a in human livers [10]. Their results suggested the indirect regulatory function of hsa-miR-148a in *CYP3A4* expression.

However, there are always controversial conclusions in clinical investigations, especially in different populations. Therefore, we asked whether their results could be replicated in Chinese Han population and whether the effect of hsa-miR-148a on PXR translation could indirectly influence *CYP3A4* expression in the population. In the present study, we found that hsa-miR-148a might not play a major role in the regulation of *PXR* or *CYP3A4* expression in human livers from Chinese Han population.

## Methods

### Liver Samples and Ethics Statement

Human liver samples from 24 Chinese Han donors were obtained in surgery from the First Affiliated Hospital of Zhengzhou University after informed consent was written. The donors included 16 males and 8 females with the age of 40.55±4.63 (mean±SEM). Among them, six had mild cirrhosis and none had Hepatitis B or C. All donors included in this study had normal liver functions and didn’t receive any preoperative medication of CYP3A4 activator or inhibitor (rifampicin, dexamethasone, propofol, etc) for 2 weeks, nor alcohol, grapefruit juice or caffeine-containing foods/beverages within 3 days before the surgery to avoid any inhibitory or inducing effects on CYP3A4 activity. The protocol complied with the Declaration of Helsinki and its subsequent revisions and was approved by the Ethics Committees of Zhengzhou University.

### Immunoblot

Total proteins were extracted using RIPA lysis buffers containing protease inhibitor PMSF. Relative protein concentrations of PXR and CYP3A4 were accessed by immunoblot using rabbit anti-PXR antibody (AP8674c; Abgent, CA) and rabbit anti-CYP3A4 antibody (ab3572; Abcam, MA), respectively, normalized to GAPDH protein concentration.

### qPCR for mRNA and miRNA

Total RNAs were extracted using TRIzol method (Life technologies, CA), followed by gel electrophoresis for quality control. Total RNAs were reversed to cDNAs using PrimeScript RT Reagent kit (Takara Bio, Dalian, China). The relative concentrations of PXR and CYP3A4 mRNA were measured by qPCR with primers (PXR: 5′-GCCCATGCTGAAATTCCACTA-3′ and 5′-GCCGATTGCATTCAATGTAGGA-3′; CYP3A4∶5′-CCAAGCTATGCTCTTCACCG-3′ and 5′-TCAGGCTCCACTTACGGTGC-3′) using FastStart Universal SYBR Green Master (Roche, IN). GAPDH mRNA was quantified as the endogenous control with primers (5′-ATCACCATCTTCCAGGAGCGA-3′ and 5′-GCTTCACCACCTTCTTGATGT-3′). For miRNA quantification, reverse PCR and qPCR were performed using miRCURY LNA™ Universal RT microRNA PCR system (Exiqon, Denmark). U6 snRNA was measured as the endogenous control. All qPCRs were run on ViiA 7 instrument (Life technologies, CA) with three replicates.

### Statistics

Linear regression, Student’s *t*-test and Spearman’s rank correlation were performed using SPSS for Windows, version 11.0 (IBM, NY). All tests were two-tailed and statistical significance was assumed at p<0.05.

## Results

### PXR mRNA Level is Correlated with PXR Protein Level in Human Livers

**Figure 1:**
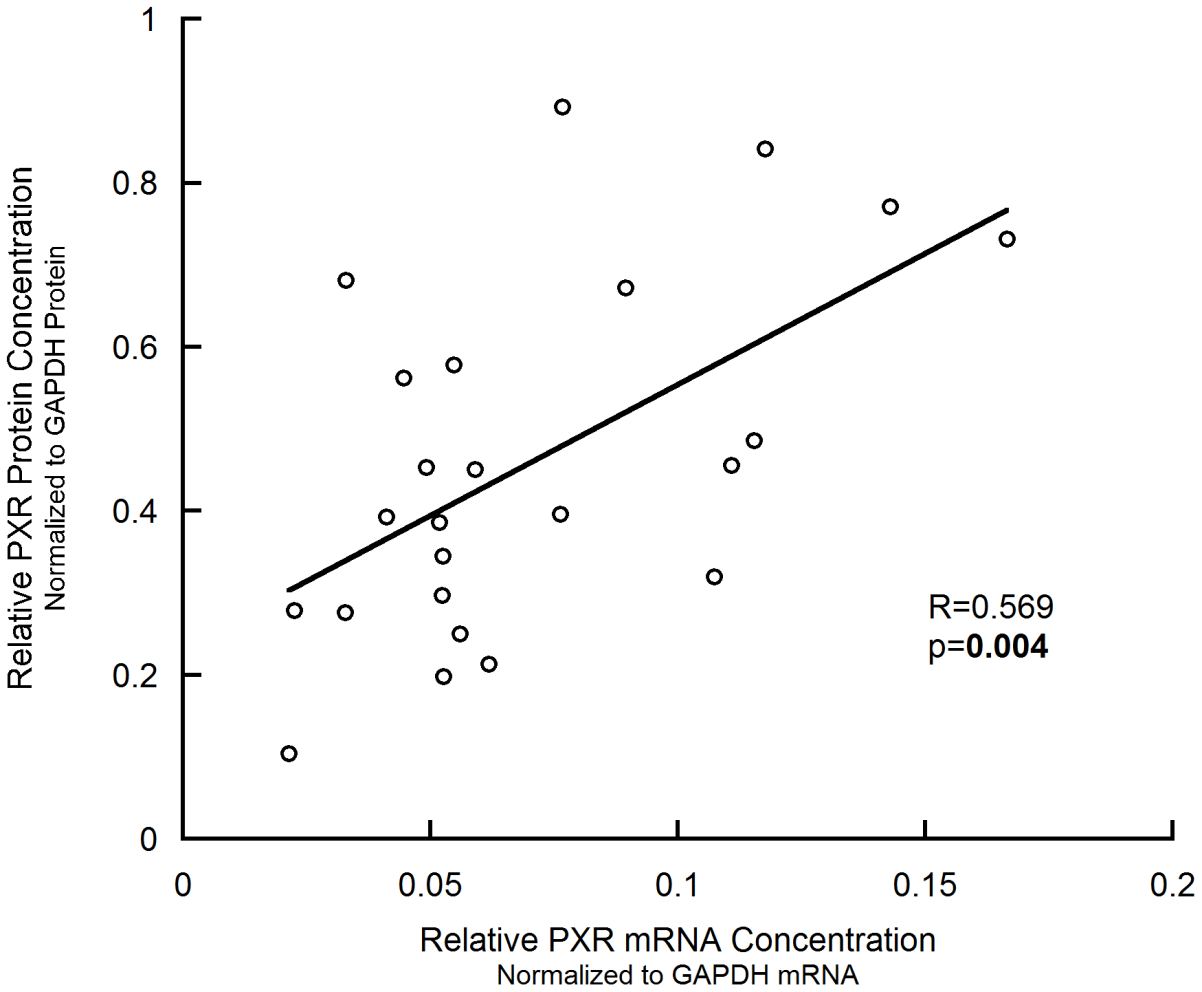
Linear correlation between PXR mRNA and protein levels in human liver samples.

We first examined the PXR mRNA level and protein level in our human liver samples (N = 24) by qPCR and immunoblot, respectively, and investigated the relationship between them. All qPCR and immunoblot data were shown in Table 1. As shown in Figure 1, significant linear correlation was observed between the PXR mRNA and protein levels (p = 0.004, R^2^ = 0.324).

**Table 1:** The relative expression of *PXR*, *CYP3A4* and hsa-miR-148a in human liver samples.

Sample No.	PXR mRNA	PXR protein	CYP3A4 mRNA	CYP3A4 protein	miR-148a
1	0.021	0.104	3.426	2.505	1.456
2	0.090	0.672	17.202	11.329	0.423
3	0.049	0.454	2.492	3.750	0.530
4	0.107	0.319	2.304	5.177	0.401
5	0.059	0.451	4.212	5.516	0.359
6	0.115	0.486	7.123	14.306	0.455
7	0.052	0.386	4.274	10.836	0.453
8	0.023	0.279	0.266	1.000	0.550
9	0.077	0.893	8.498	16.002	0.285
10	0.053	0.198	7.177	7.928	0.406
11	0.056	0.250	2.764	2.329	0.665
12	0.053	0.345	7.856	16.663	0.497
13	0.143	0.771	15.766	19.160	0.467
14	0.041	0.393	4.652	6.198	0.123
15	0.111	0.456	9.540	8.236	0.701
16	0.033	0.276	1.335	0.958	0.627
17	0.167	0.732	8.721	14.018	0.620
18	0.052	0.297	5.657	6.700	0.559
19	0.062	0.214	9.563	10.117	0.726
20	0.076	0.396	4.290	4.353	1.838
21	0.118	0.841	10.731	9.453	1.735
22	0.055	0.578	8.798	12.492	1.590
23	0.045	0.562	11.558	13.107	0.778
24	0.033	0.681	1.402	1.706	1.738

The relative RNA levels of mRNAs and miRNA were measured using qPCR with the normalization to the GAPDH mRNA level and the U6 snRNA level, respectively. The relative protein levels were measured using immunoblot with the normalization to the GAPDH protein level.

### PXR Protein Level is Correlated with CYP3A4 mRNA Level in Human Livers

**Figure 2:**
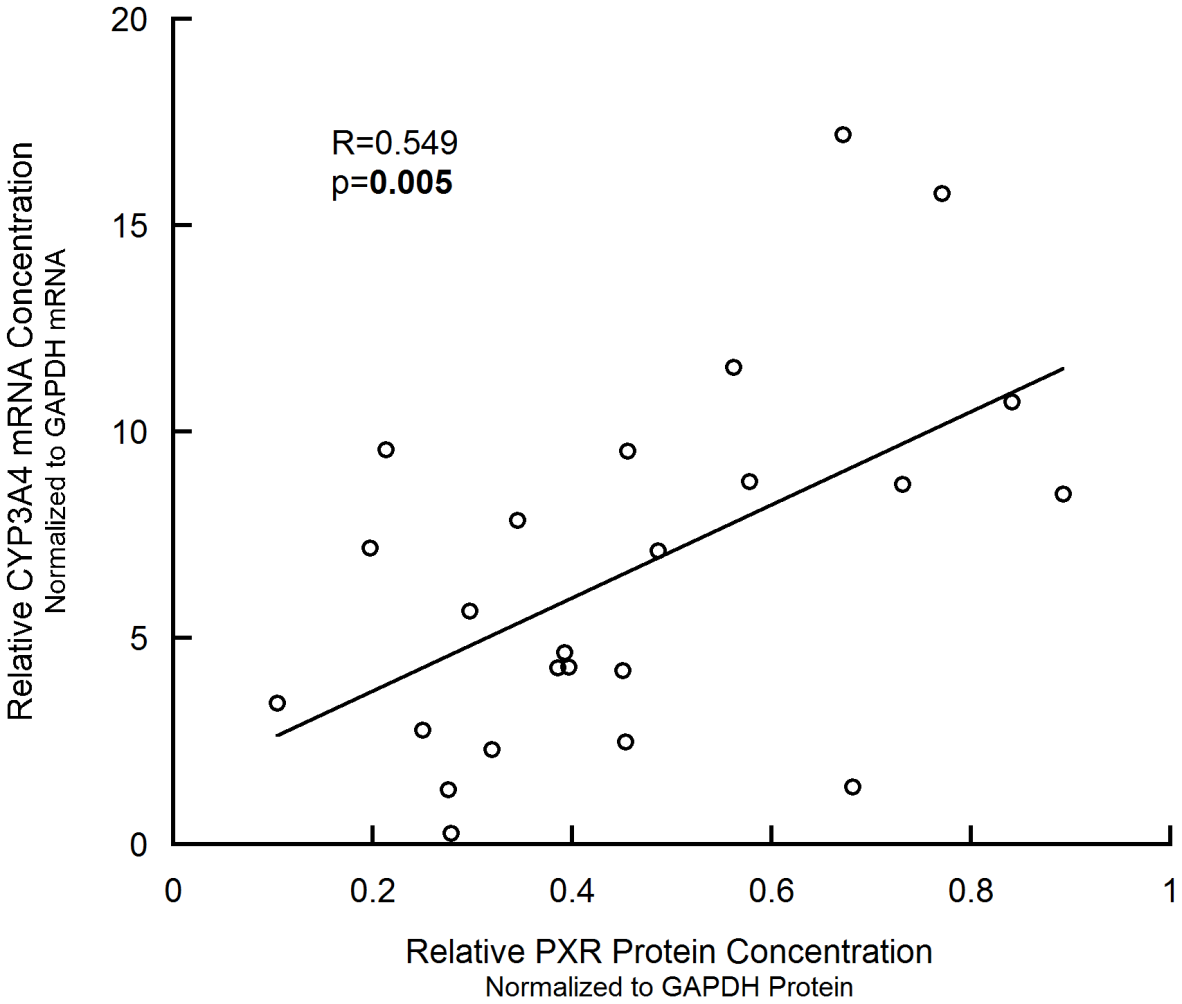
Linear correlation between PXR protein and CYP3A4 mRNA levels in liver samples.

The CYP3A4 mRNA level in human liver samples was measured by qPCR, and was investigated about its relationship with the PXR protein level. As shown in Figure 2, significant linear correlation was observed between the PXR protein level and CYP3A4 mRNA level (p = 0.005, R^2^ = 0.301).

### Hsa-miR-148a did not Affect Expression of *PXR* in Chinese Han Population

**Figure 3:**
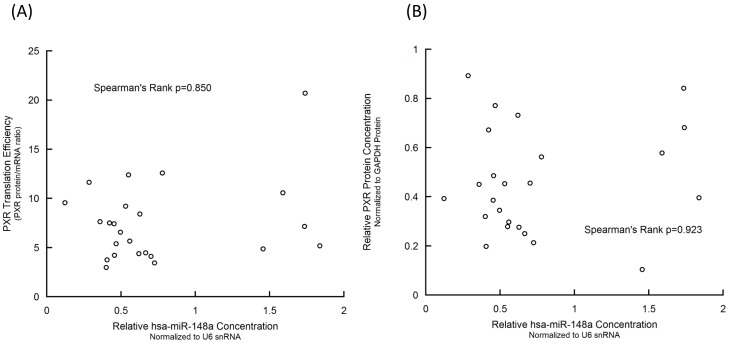
Hsa-miR-148a was not correlated with the translational efficiency (A) or protein level (B) of PXR.

We calculated the PXR protein/PXR mRNA ratio as an index of the translational efficiency of PXR, according to Takagi et al. [10]. There was no significant correlation between the translational efficiency of PXR and the hsa-miR-148a level in liver samples using Spearman’s rank method (p = 0.850) or linear regression (p = 0.217), as shown in Figure 3A. The hsa-miR-148a level was not significantly correlated with PXR protein level either (Spearman’s rank p = 0.923; Figure 3B).

### Hsa-miR-148a did not Show Influence on the Expression of *CYP3A4* in Chinese Han Population

**Figure 4:**
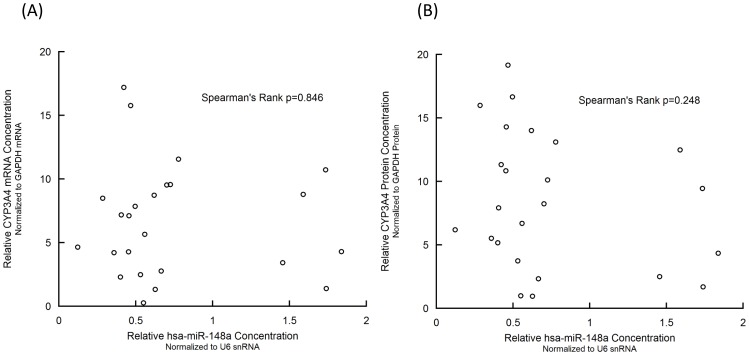
No correlation between hsa-miR-148a level and CYP3A4 mRNA (A) or protein (B) levels in livers.

To investigate whether hsa-miR-148a could indirectly regulate *CYP3A4* transcription, we examined the relationship between hsa-miR-148a level and CYP3A4 mRNA level. As shown in Figure 4, hsa-miR-148a level in human livers was not significantly in Spearman’s rank correlation with CYP3A4 mRNA level (p = 0.848) or protein level (p = 0.248).

### Hsa-miR-148a Grouping or Cirrhosis Grouping didn’t Associate with the Expression of *PXR* or *CYP3A4*

The relative expression of hsa-miR-148a seems to aggregate into two groups with a split point as 1.0 as shown in Figure 3. We compared the relative expression of *PXR* and *CYP3A4* between high and low hsa-miR-148a expression groups. As shown in Table 2, there was no significant difference between these two groups. We also analyzed and found that mild cirrhosis status had no significant influence on PXR or CYP3A4 levels (Table 2). To be noticed, mild cirrhosis status didn’t show significant correlation with hsa-miR-148a expression (p = 0.472).

**Table 2:** The expression of *PXR*, *CYP3A4* and hsa-miR-148a in groups according to hsa-miR-148a level and cirrhosis status.

	Hsa-miR-148a level			Mild cirrhosis		
	High	Low	p-value	Yes	No	p-value
N	5	19		6	18	
PXR mRNA level	0.061±0.017	0.073±0.009	0.533	0.071±0.014	0.070±0.010	0.946
PXR protein level	0.520±0.127	0.444±0.046	0.492	0.428±0.107	0.470±0.048	0.687
PXR translational efficiency	9.70±2.931	6.91±0.704	0.174	6.301±1.268	7.892±1.010	0.413
CYP3A4 mRNA level	5.729±1.740	6.893±1.055	0.611	5.973±1.234	6.876±1.139	0.673
CYP3A4 protein level	6.102±2.090	9.123±1.249	0.270	9.017±2.431	8.319±1.249	0.789
hsa-miR-148a level	1.672±0.067	0.507±0.037	9.3E-13	0.616±0.178	0.794±0.126	0.472

Data expressed as mean±SEM. Homoscedasticity between groups was verified using Levene’s test.

## Discussion

PXR is a major transcription factor of *CYP3A4* gene, and regulates *CYP3A4* expression. In 2008, Takagi et al. found that hsa-miR-148a could bind to the 3′-UTR region of *PXR* and influence its expression both *in vitro* and *in vivo*. However, our results suggest that hsa-miR-148a may not be involved in the regulation of *PXR* expression or further *CYP3A4* expression.

The significant correlation between PXR protein level and CYP3A4 mRNA level is in line with the consensus that PXR is a transcription factor of *CYP3A4*, also consistent with Takagi et al.’s results [10]. Nevertheless, the linear correlation between PXR mRNA and protein levels is inconsistent with Takagi et al.’s results. They explained the non-significant correlation as the involvement of post-transcriptional regulation. Our significant correlation result, however, still couldn’t rule out the involvement of miRNA’s regulation, considering the R^2^ value is small (R^2^ = 0.324).

As a replication study, we tried to correlate the hsa-miR-148a level with the translational efficiency of PXR in our liver samples. The correlation was non-significant no matter using traditional linear regression or the Spearman’s rank method as Takagi et al. used. To confirm our non-significant result, we also correlated the miRNA level and the PXR protein level. The correlation was still statistically non-significant. Furthermore, hsa-miR-148a did not show significant effect on *CYP3A4* expression either.

It is a general problem that the clinical correlation study in one population cannot be replicated in another population. Several differences may contribute to the inconsistency of the influence of hsa-miR-148a between Takagi et al.’s conclusion and ours. (1) All our samples were from Central China, while Takagi et al.’s samples were Japanese. The ethnic difference might be the major factor contributing to the different conclusions. (2) In our samples, PXR protein level showed linear regression with PXR mRNA level, which was non-significant in Takagi et al.’s result. This may weaken the relative impact of miRNAs on *PXR* expression. (3) The techniques to quantify the concentration of miRNA were different between the two studies. In this study, we used miRCURY LNA™ technology to make sure better sensitivity and more importantly higher specificity. (4) The regulation of *PXR* expression is complex and involves multiple signaling mechanisms, while the effect of hsa-miR-148a on the regulation of *PXR* expression might be weak. Besides, both these two studies had only small sample sizes, which impaired the statistical power of the conclusion. To offset the probability of false-negatives caused by limited sample size, we re-evaluated the alpha threshold using G*Power version 3.0 [11]. With a medium size effect (f^2^ = 0.2), alpha threshold should be set as 0.191 to achieve power = 0.8. The non-significant correlation of hsa-miR-148a level with PXR and CYP3A4 expression still exceeded this more stringent threshold.

In summary, we failed to replicate the significant correlation between liver hsa-miR-148a level and the translational efficiency of PXR in Chinese Han population. Thus, our results didn’t support the significant effect of hsa-miR-148a in *PXR* expression in human livers. Further replications with larger sample sizes and different ethnic populations were warranted to fully elucidate the exact role of hsa-miR-148a in the regulation of *PXR* expression and then *CYP3A4* transcription.
